# Supplementary material for: ATP requirements for growth reveal the bioenergetic impact of mitochondrial symbiosis
Source: Biochim Biophys Acta Bioenerg. Author manuscript; Available in PMC 2025 Aug 1. (PMC7617979; doi:10.1016/j.bbabio.2025.149564)
Supplement: Table S1 [file EMS207424-supplement-Table_S1.pdf]

Supplemental Table S1

Calculated costs for synthesizing an amino acid (one letter code) in *Methanococcus* using NH_4^+ or N_2 as nitrogen source. Amino acid frequencies were calculated from the *Methanococcus thermolithotrophicus* genome sequence. As shown in Figure 1 of the main text, the estimation of amino acid frequencies in protein from the genome sequence and chemical method for *E. coli* correspond well.

Table S1: ATP requirements for amino acid synthesis in a H_2-dependent methanogen grown on NH_4^+ or N_2

AA	mmol per 524 mg protein	Precursor, cost [ATP][c]	additional ATP	NH_4^+ ATP per amino acid	NH_4^+ mmol ATP per g cells	N_2 ATP per amino acid	N_2 mmol ATP per g cells
A	0.255	Pyr [0]	N [1]	1	0.254	9	2.29
C	0.066	3PGA [2]	N [1]	3	0.199	11	0.731
D	0.278	OA [3]	N [1]	4	1.115	12	3.35
E	0.397	2OG [4]	N [1]	5	1.984	13	5.15
F	0.183	3PGA [3] (2x)	Chor. [1] N [1]	8	1.464	16	2.93
G	0.318	3PGA [2]	N [1]	3	0.955	11	3.50
H	0.076	PRPP [5]	2 PPi [4] N [3]	12	0.916	36	2.75
I	0.464	Pyr [0] Thr [7]	N [1]	8	3.714	16	7.43
K[a]	0.464	Pyr [0] Asp-SA [5]	Succ-CoA [1] N [1]	7	3.249	23	10.7
L	0.431	Pyr [0] Ac-CoA [0]	N [1]	1	0.430	9	3.87
M	0.119	Pyr [0] Asp-SA [5]	Succ-CoA [1] N [1]	7	0.833	15	1.78
N	0.274	Asp [4]	PP_i [2] N [1]	7	1.916	23	6.30
P	0.165	Glu [5]	γ-Glu-P [1]	6	0.988	14	2.30
Q	0.080	Glu [5]	N via γ-Glu-P [1]	6	0.482	22	1.77
R	0.164	Glu [5] CAP [1]	N-AcGlu-P [1] N [2] PPi [2]	11	1.800	43	7.04
S	0.253	3PGA [2]	N [1]	3	0.758	11	2.78
T	0.219	Asp [4]	Asp-P [1] HS-P [1]	6	1.315	14	3.07
V	0.331	2 Pyr [0]	N [1]	1	0.331	9	2.98
W[b]	0.032	3PGA [3] (2x); PRPP [5]	Chor [1] N [1] Ser [3]	15	0.480	31	0.95
Y	0.191	3PGA [3] (2x)	Chor [1] N [1]	8	1.528	16	3.06
	4.761				24.71		74.77

The pathways are taken from Lengeler [78]. Abbreviations: Asp-P aspartyl-4-P; HS-P homoserine-P; Chor. chorismate; Asp-SA aspartate semialdehyde; Ac-CoA. Acetyl-CoA; PP_i Pyrophosphate; N-AcGlu-P *N*-acetylglutamyl-P; Chor chorismate; γ-Glu-P γ-glutamyl phosphate. [a] Methanogens use the diaminopimelate pathway [112]. [b] in the final step, glyceraldehyde-3-phosphate is released, which yields 1 ATP gain, hence 15 instead of 16. [c] from Figure 2 of the main text. For aromatic amino acids, methanogens synthesize chorismate from 6-deoxy-5-ketofructose [113], derived from two molecules of 3PGA, as opposed to 3-deoxy-arabino-heptulosonate (in *E. coli* derived from E4P and PEP) such that but the ATP expense is increased by one ATP per amino acid relative to the classical shikimate pathway. Cysteine synthesis also differs from the bacterial pathway in methanogens [114], but without difference in ATP expense.
